# Unveiling autism spectrum disorder for the pediatrician

**DOI:** 10.1016/j.jped.2025.101458

**Published:** 2025-11-03

**Authors:** Marcio Moacyr Vasconcelos, Luciana Gonçalves Azevedo Vasconcelos, Adriana Rocha Brito

**Affiliations:** a Universidade Federal Fluminense (UFF), Departamento Materno-Infantil, Niterói, RJ, Brazil; b Associação Brasileira Beneficente de Reabilitação (ABBR), Departamento Infanto-Juvenil, Rio de Janeiro, RJ, Brazil

**Keywords:** Etiology, Genetics, Epigenetics, Pathophysiology, Diagnosis, Management

## Abstract

**Objectives:**

To review the state of the art in autism spectrum disorder (ASD), including its etiologic puzzle, clinical features, pathophysiologic mechanisms, differential diagnosis, and therapeutic management.

**Data sources:**

A search for papers published over the past 10 years in the databases PubMed-MEDLINE, Cochrane Library, and SCIELO was performed using the following terms: “autism” and “clinical features”, “differential diagnosis”, “pathophysiology”, or “management”. The search yielded 3240, 590, 6904, and 5023 papers, respectively. A total of 120 most relevant papers were selected based on their title and abstract content.

**Data synthesis:**

The current prevalence of ASD is 1 in every 31 eight-year-old children. A genetic defect is found in 10–20% of individuals with ASD. Environmental risk factors that increase the likelihood of ASD include advanced parental age and maternal health conditions. Epigenetic mechanisms may play a crucial role in the interplay between genetic and environmental factors in the pathogenesis of ASD. In addition to language delay, pediatricians should monitor and screen for several early signs of ASD. Differential diagnosis is complex because several neurodevelopmental conditions show clinical features that overlap with ASD. Medications may be used to treat comorbid conditions.

**Conclusions:**

Management is based on a multidisciplinary team, and pediatricians are in a unique position to coordinate this team, given the trustworthy relationship they have with patients and their families.

## Introduction

Autism spectrum disorder (ASD) prevalence rose from 1 in every 152 to 1 in every 31 eight-year-old children in just two decades, according to the Autism and Developmental Disabilities Monitoring (ADDM) Network biennial surveys [1,2]. This marked elevation has turned ASD into a commonplace, if not a daily concern, in the practice of pediatricians. ASD results from a deranged neurodevelopment leading to a recognizable behavioral and cognitive syndrome, the core deficits of which spread over two domains: social communication-interactions and restrictive, repetitive patterns of behavior, interests, and activities [3].

ASD is typically diagnosed during the first three years of life with a spectrum of symptom severity that is classified in levels of support 1 (mild), 2 (moderate), or 3 (severe). There are significant gender disparities, as boys are affected 4–5 times more frequently than girls [4,5].

The advent in 2013 of the fifth edition of the Diagnostic and Statistical Manual of Mental Disorders (DSM-5) [5] simplified ASD diagnosis, as one no longer needs to struggle with alternative diagnoses, such as Asperger’s syndrome, childhood disintegrative disorder, or pervasive developmental disorder not otherwise specified. DSM-5 adopted a dimensional approach to diagnosis, as opposed to DSM-IV.

The goal of this paper is to review the state of the art in ASD, including its etiologic conundrum, clinical features, pathophysiologic mechanisms, differential diagnosis, and therapeutic management.

## Etiology

Knowing that most children with ASD lack an identifiable cause should not discourage pediatricians from investigating the etiology of their condition. Understanding the cause can help explain the condition to parents, predict comorbidities, guide treatment decisions, estimate recurrence risks, and form the basis for personalized therapy plans [3].

Genetic and environmental factors influence the etiology of ASD. Twin and family studies showed that genetic factors play a significant role, with an estimated heritability of 40–90 %. Large-scale genomic studies have identified hundreds of genetic defects, including *single-nucleotide variants* (SNVs) and genomic *copy number variants* (CNVs), in association with ASD [6]. *ADNP, CHF8, FMR1, SHANK3* and *PTEN* are among the genes frequently reported in association with ASD (Table 1) [7].

**Table 1:** Genes associated with autism spectrum disorder.

Gene	Locus	Inheritance	Clinical features
*ADNP*	20q13.13	AD	ADNP syndrome – ID, notched eyelid, absent or severely delayed speech
*ARID1B*	6q25.3	AD	Coffin-Siris syndrome – ID, facial hypertrichosis, visual impairment
*CACNA1C*	12p13.33	AD	Timothy syndrome - Long QT interval, syndactyly
*CHD8*	14q11.2	AD	ID, macrocephaly, epilepsy, sleep disturbances
*CNTNAP2*	7q35-q36.1	—	Mild to moderate ID, epilepsy, speech abnormalities, cortical dysplasia
*DYRK1A*	21q22.13	AD	ID, microcephaly
*FMR1*	Xq27.3	XLD	ID, Fragile X syndrome
*FOXP1*	3p13	AD	ID, language impairment
*FOXP2*	7q31.1	AD	Developmental verbal dyspraxia
*GRIN2B*	12p13.1	AD	ID, epileptic encephalopathy
*MECP2*	Xq28	XLD	ID, Rett syndrome
*NLGN4*	Xp22	XL	ID, dysarthria, motor tics
*NRXN1*	2p16.3	AR	ID, schizophrenia, mild facial dysmorphism
*PTCHD1*	Xp22.11	XLR	ID, motor tics
*PTEN*	10q23.31	AD	Mild to moderate ID, macrocephaly, Cowden syndrome
*RELN*	7q22.1	AD	Epilepsy
*RPL10*	Xq28	XLR	ID, progressive microcephaly
*SCN2A*	2q24.3	AD	Epileptic encephalopathy
*SHANK2*	11q13.3-q13.4	—	Mild to moderate ID
*SHANK3*	22q13.33	AD	Phelan-McDermid syndrome – tall stature, epilepsy, absent or severely delayed speech, moderate to severe ID
*SYNGAP1*	6p21.32	AD	ID, epilepsy
*TSC1 TSC2*	9q34.13 16p13.3	AD	Tuberous sclerosis, epilepsy

AD, autosomal dominant; AR, autosomal recessive; ID, intellectual disability; XL, X-linked; XLD, X-linked dominant; XLR, X-linked recessive.

*Source*: Adapted from reference 7.

It is currently estimated that 10–20 % of people with ASD have a *de novo* rare point mutation or CNV contributing to their phenotype. Certain clinical features may increase this contribution, such as female gender, intellectual disability, seizures, and having multiple unaffected siblings. The odds ratio of an ASD diagnosis is approximately 16.0 for children who have an older sibling with ASD [8].

A study using whole exome sequencing (WES) in a sample of 35,584 individuals, including 11,986 with ASD, identified 102 genes potentially implicated in autism. Expression of these genes is enriched in both excitatory and inhibitory neuronal lineages, favoring the hypothesis of an excitatory-inhibitory imbalance underlying ASD [9]. Another study in a cohort of 63,237 individuals found 72 genes highly associated and 185 genes moderately associated with ASD. The researchers concluded that, compared with genes linked with developmental delay (DD) and schizophrenia, genes expressed at earlier stages of cortical development, such as progenitor genes, broadly show greater DD enrichment, while those expressed later, like maturing neurons, tend to be implicated with ASD. This finding “is consistent with the expectation that earlier and more generalized impairment leads to severe global DD while later, neuron-specific impairment affects more isolated developmental domains, such as social interaction and the presence of repetitive behaviors and/or interests that typify ASD” [10].

Several studies on the genetic architecture of ASD have shown an excess of loss-of-function mutations in hundreds of genes, which code for proteins involved in synaptic formation, transcriptional regulation, and chromatin remodeling pathways. It is hypothesized that altered chromatin dynamics and transcription may also disrupt synaptic function [11].

CNVs consist of deletions and duplications of specific DNA segments. They are more common in simplex families, where only one proband is affected with ASD [12]. They are also known to be abundant in the human brain, contributing to human diversity, as well as to various medical conditions, including neurodevelopmental delay, autism spectrum disorder, and neuropsychiatric disorders [13]. Frequently replicated CNV regions have included 1q21.1, 3q29, 7q11.23, 16p11.2, 15q11.2–13, and 22q11.2. Adding complexity to their understanding, autism-associated CNVs are highly pleiotropic, that is, many CNVs are also associated with intellectual disability, schizophrenia, and ADHD [13].

Lastly, there seem to be environmental risk factors that increase the likelihood of ASD, such as advanced parental age (maternal age ≥ 40 years and paternal age ≥ 50 years); maternal health conditions during pregnancy, e.g., infection, autoimmune disease, diabetes, thyroid disorders, exposure to sex hormones or toxic substances, and use of certain medications, like valproate; and maternal psychiatric conditions [14,15]. A study on maternal valproate therapy for epilepsy or bipolar disorder provided strong evidence in favor of its association with ASD and other neurodevelopmental impairment. Gestational and perinatal events leading to ischemia, hypoxia, or trauma have also been strongly linked to ASD [16].

Importantly, a large body of scientific evidence disproves allegations that vaccines cause ASD. The ill-founded link between vaccines and ASD has led to increased vaccination refusal among parents, resulting in outbreaks of preventable diseases like measles [17,18].

## Pathophysiology

The genetic defects described above are usually involved in multiple functions in many cerebral regions, hindering attempts to establish a single pathophysiological mechanism for ASD. Nevertheless, “most proteins encoded by autism risk genes are involved in either synaptic structure and function or chromatin modification and regulation of gene expression” [8]. In addition, studies on excitatory glutamatergic neurons during cortical development have suggested specific molecular pathways and circuits in autism.

One study examining 10 genes with the strongest statistical association with ASD found that they were expressed in the telencephalon at critical points of prefrontal cortex development. Additionally, estrogen mitigates the effects of ASD risk gene disruption, a likely explanation for the male gender preponderance in autistic populations [19].

Epigenetic mechanisms, such as DNA methylation, histone modifications, and microRNA dysregulation, may play a crucial role in the interplay between genetic and environmental factors in the pathogenesis of ASD. They change gene expression, for example, transcriptional silencing, without interfering with the DNA sequence. In addition, they have been shown to influence activation of immune responses, increasing susceptibility to ASD [20].

Neuronal migration disturbances during fetal development, demonstrated by imaging studies, are another neurobiological pathway leading to ASD through cerebral white matter disorganization and resultant connectivity impairment [4].

A wide variety of immunologic disorders have been reported in patients with ASD, including altered cytokine profiles, T cell dysfunction, reduced number of B‑cells, increased amount of NK cells, and the presence of autoantibodies to many targets. The resultant neuroinflammation produces microglial activation, which, when excessive, can damage healthy synaptic connections. Intense microglial activation was observed in the cerebral cortex, white matter, and especially the cerebellum of autistic patients [21].

Prospective studies have shown typical profiles of interest in faces and eyes at age 6 months in infants who will receive a diagnosis of ASD. Research on electroencephalography has revealed subtle findings that implicate synaptic signaling pathways, potentially impacting infantile development [8].

It is unlikely that a deterministic model will fully explain the pathophysiology of ASD [8], as a series of interactions among several brain regions is required to disrupt the inherently complex human development.

## Clinical picture

The first clinical sign to raise ASD suspicion is often delayed language development. At age 18 to 24 months, a speech delay not accompanied by compensatory pointing or gesturing strengthens one’s index of suspicion. A child older than 24 months with echolalia as the only language is likely to receive a diagnosis of ASD [22].

In addition to language delay, pediatricians should monitor and screen for several early signs of ASD (Table 2).

**Table 2:** Early clinical signs of autism spectrum disorder.

*Speech and language delay*. Vocabulary restricted to a few words at age 18–24 months; one third of cases lose previously acquired words, i.e., regression; impaired speech prosody; echolalia and/or jargon speech beyond 2–2.5 years of age.
*Deficits in social interaction*. Unable to understand others’ nonverbal cues or emotions; lack of reciprocity in an attempt at conversation; awkward attempts to initiate or respond to social interaction.
*Deficits in nonverbal communication*. No pointing and no waving goodbye at 18 months; precarious or nonexistent eye contact; lack of facial expressions.
*Impairments in play*. No symbolic play; banging or throwing toys instead of using them appropriately; preference for unusual objects; hyperfocus on small parts, e.g., a car door or wheel.
*Abnormal movements and behaviors*. Stereotypic movements, e.g., hand flapping; wandering or moving around in circles; finger wiggling close to one’s eyes.
*Lack of joint attention*. Inability to share a focus of interest with another person, e.g., not bringing a toy to their parents, or not sharing a toy with peers.
*Strong preference for sameness*. Rigid adherence to routines and rituals; an unexpected change may trigger anxiety or irritability.
*Sensory dysfunction*. Hyper or hyporeactivity to noise, smell, or texture; apparent indifference to pain or temperature.

*Source*: Adapted from references 5 and 24.

“Importantly, there is no one behavioral feature that is universal to all individuals with ASD, just as there is no symptom that is entirely specific to ASD. This significantly complicates diagnosis, because children with various neurodevelopmental disorders or psychiatric diagnoses may present with symptoms that appear very similar to ASD …Thus, it is only after careful consideration of a child’s complete developmental and behavioral profile that a diagnosis of ASD should be considered” [23]. On the other hand, there is no single behavior that, once present, rules out a diagnosis of ASD.

At which point does a specific variant behavior become a clinical feature of autism spectrum? To answer this question, pediatricians must familiarize themselves with the core features listed in DSM-5 and apply the prerequisite that an ASD symptom causes “clinically significant impairment in social, occupational, or other important areas of current functioning” [5].

## Diagnosis

Diagnosis is usually based on detailed developmental history, clinical judgment, and the use of standardized diagnostic instruments [24]. It is crucial to keep in mind that, as opposed to many other pediatric diagnoses, it is impossible to diagnose ASD based on the results of a given test. There are no specific biomarkers, and no behavioral test is sufficiently accurate by itself to identify ASD. A word of caution regarding standardized diagnostic instruments: although they may help the diagnostic process, their misuse, including overreliance on scores and classifications, is a likely cause of the current high rates of misdiagnosis [25].

Trained pediatricians can confidently suspect ASD based on their patients’ behavioral presentation, according to the criteria defined by DSM-5. Despite the significant heterogeneity that exists between and within individuals across development, they will be able to detect the core diagnostic features of autism, which encompass social interaction, communication, and restricted, repetitive, or sensory behaviors [8].

Notwithstanding, a formal ASD diagnosis should not rely solely upon a child’s behavioral presentation; instead, it is advisable to request pertinent information from their parents as well as other caregivers, teachers, and therapists [26].

The pediatrician can facilitate early identification by using a validated developmental screening test. Universal screening for ASD at 18- and 24-months pediatric visits utilizes a standardized tool. The most widely used is the Modified Checklist for Autism in Toddlers (M-CHAT), which is validated for the age range 16 to 30 months, and is freely available for download. Its most recent version is the M-CHAT Revised with Follow-up (MCHAT-R/F) [24]. The M-CHAT follow-up interview was established to reduce its high false-positive screening results. In addition to periodic screening, pediatric healthcare professionals should continually surveil children at each pediatric health supervision visit throughout the first 5 years of life, with special attention to developmental concerns expressed by parents, teachers, or other healthcare professionals [27]. If a child raises suspicion of ASD during screening or surveillance, it is appropriate to refer them for a comprehensive ASD evaluation and concomitantly for early intervention [24]. Siblings of children with ASD warrant intensified surveillance due to their elevated risk for ASD and other neurodevelopmental disorders [28].

For some individuals, the diagnostic criteria of ASD are not met until mid-childhood, adolescence, or even adulthood. In addition, diagnosis beyond early childhood can occur even in those children who remained under close developmental monitoring [8].

## Differential diagnosis

ASD differential diagnosis is particularly complex because several neurodevelopmental conditions show clinical features that overlap with ASD, not to mention they may be co-occurring. Clinicians should remember that most individuals diagnosed with ASD have comorbidities [23]. For example, at least one comorbidity was present in 74 % of 42.569 individuals with ASD, including attention deficit/hyperactivity disorder (ADHD) in 35.3 %, learning disability in 23.5 % and intellectual disability in 21.7 % [29].

Table 3 lists the most common conditions that need to be differentiated from ASD.

**Table 3:** Distinguishing clinical features in the differential diagnosis of autism spectrum disorder.

	Features against ASD *	Features in favor of ASD *
ADHD *	Intact social communication and interactions	Deficit in nonverbal communication
Language disorders	Pointing; sharing attention; coordinating gaze, facial expressions, and gestures	Lack of imitation and limitations in pretend play
Selective mutism	Normal early development Appropriate communication in certain settings	Restrictive/repetitive patterns of behavior.
Intellectual disability	No discrepancy between social communication and other intellectual skills	Deficits in social communication and interactions relative to the developmental level
Stereotypic movement disorder	Normal development	Deficits in social communication
Social pragmatic communication disorder	Isolated deficits in pragmatic language	Presence of restricted and repetitive behaviors
Anxiety disorders	Age of onset > 5 years	Early age of onset
Depression disorders	Later age of onset; often episodic	Suggestive developmental history
Obsessive-compulsive disorder	Compulsions may be distressing	Repetitive behaviors are enjoyed
Schizophrenia	Psychotic features; typical onset in late adolescence or early adulthood	Lack of a preceding normal or near-normal development
Rett syndrome	Female gender Improved social communication after 4 years of age	Male gender All diagnostic criteria are met

*Source*: Adapted from references 5 and 23.

⁎ ADHD, attention deficit/hyperactivity disorder; ASD, autism spectrum disorder.

Hearing screening is an integral part of the ASD diagnostic workup. Once a diagnosis is established, a genetic evaluation is pertinent, including fragile X testing and chromosomal microarray. Additional workup, such as metabolic testing, electroencephalogram (EEG), neuroimaging, and other studies, is guided by history and physical examination [3].

## Management

Adequate management of a child affected with ASD relies substantially on the primary care pediatrician, as it requires immediate suspicion triggered by the early clinical signs and symptoms described above, as well as responsible consideration of parental concerns about a child’s development in the first years of life. In the current era, there is no room for playing down parental concerns and postponing a multidisciplinary team assessment. Therapeutic interventions should never wait for a definitive diagnosis [24]. The pediatricians are in a unique position to coordinate this multidisciplinary team, given the trustworthy relationship they have with patients and their families.

One should implement management with the help of a multidisciplinary team, which consists of, but is not restricted to, a speech therapist, occupational therapist, special educator, and psychologist [30]. Behavioral interventions, such as Early Intensive Behavioral Intervention with ABA (Applied Behavioral Analysis) and Early Start Denver Model (ESDM), are well-supported by evidence. They have proven to benefit social-emotional functioning and challenging behaviors [31,32]. Speech therapy, sometimes supplemented with technology-based interventions such as augmentative and alternative communication (AAC), and occupational therapy incorporating Ayres sensory integration may also offer benefits for social communication and social-emotional skills [31,33].

The goal of therapy is to improve an individual’s function and well-being. A successful plan must address communication, social skills, adaptive skills, sensory processing, behavior, and mental health [17,32]. Additionally, there is no “one size fits all” approach to treatment. The therapeutic plan should consider the child’s current strengths and challenges, as well as the family's priorities, goals, and available resources [32].

No medications have demonstrated efficacy for the core clinical features of ASD [17]. One exception to this rule may be cannabidiol (CBD) [34]. However, pharmacologic agents, such as risperidone and aripiprazole, can treat comorbid conditions, e.g., ADHD, sleep disturbances, mood disorders, and anxiety, and can help relieve irritability, aggression, and emotional outbursts [35,36]. Table 4 lists the most commonly used medications in these patients.

**Table 4:** Medications potentially useful for children with autism spectrum disorder.

Medication	Indications	Precautions or Contraindications	Side effects
Risperidone	Irritability Aggression Emotional dysregulation	Dyslipidemia Hyperprolactinemia Prolonged QT interval	Weight gain, drowsiness, fatigue, GI* symptoms, urinary retention, extrapyramidal features
Aripiprazole	Irritability Aggression Emotional dysregulation	Dyslipidemia Hyperprolactinemia Prolonged QT interval	Weight gain, drowsiness, fatigue, GI* symptoms, extrapyramidal features
Clonidine	ADHD Insomnia	Hypotension	Dry mouth, drowsiness, dizziness, emotional/tearful
Psychostimulants, e.g., methylphenidate, lisdexamfetamine	ADHD	Glaucoma Anxiety Tourette syndrome Hypertension	Anorexia, irritability, insomnia, headache, GI* symptoms, hypertension
Atomoxetine	ADHD	Glaucoma Hypertension Hyperthyroidism	Headache, diurnal somnolence, GI* symptoms, anorexia, hypertension
SSRIs*	Anxiety/depression Restricted, repetitive behaviors	Glaucoma Liver or renal dysfunction	Headache, insomnia, nervousness, drowsiness, GI* symptoms
Melatonin	Delayed sleep phase Insomnia	Narcolepsy	Drowsiness, headache
N‑acetylcysteine	Irritability Stereotypic behaviors	Asthma	GI* symptoms, bronchospasm, drowsiness
Cannabidiol	Behavioral symptoms	Still considered experimental Liver dysfunction	Dizziness, insomnia, irritability, GI* symptoms, weight gain, increased aggression

*Sources*: Adapted from references 17 and 34–36.

⁎ GI, gastrointestinal; SSRIs, selective serotonin reuptake inhibitors, e.g., fluoxetine, sertraline, escitalopram.

Controlled trials and cohort studies on the use of CBD or CBD-rich cannabis have demonstrated promising improvements in behavioral symptoms, social responsiveness, and communication [34,37,38]. Their side effects included somnolence, decreased appetite, and increased aggression. Future studies should investigate the risk-benefit ratio, mechanism of action, and optimal dosages of CBD [39].

A Cochrane systematic review found no evidence for the beneficial effect of heavy metal chelation therapy [40]. Although individuals with ASD have frequently sought complementary, alternative, and integrative Medicine, there is no high-quality evidence to support their use [41].

A prospective, double-blinded, randomized, placebo-controlled clinical trial on corticosteroid therapy for ASD found a beneficial effect on language function, mainly in children under five years of age with a history of developmental regression [42]. Although there have been many anecdotal reports of benefit, for example, in children with EEG spikes localized to the peri‑sylvian region, inferior frontal, or peri‑rolandic region as opposed to those with multifocal or widespread spike abnormalities on EEG [43], there is a need for additional studies with larger sample sizes and more homogeneous patient samples to clarify the potential value of corticosteroids in ASD treatment.

## Outcome

When assisting autistic children and families in navigating their developmental journey, pediatricians should know that a small percentage of patients eventually outgrow their condition, the so-called optimal outcome [44]. It is currently unknown whether this result is due to early intervention or represents a distinct subtype of autism [8].

Many countries, including Brazil, have complied with the United Nations determination that ASD must be recognized as a disability requiring mandated services. However, “there remain enormous challenges to ensure people with autism and their families the same opportunities for happiness, health, and community participation” [45].

## Funding source

None.

## Conflicts of interest

The authors declare no conflicts of interest.

## Data Availability

The data that support the findings of this study are available from the corresponding author.
